# Acute Lymphoblastic Leukemia in a Man Treated With Fingolimod for Relapsing Multiple Sclerosis

**DOI:** 10.1177/2324709615575551

**Published:** 2015-03-09

**Authors:** Stanley Cohan, John Godwin, Leah Gaedeke

**Affiliations:** 1 Providence Health & Services, Portland, OR, USA

**Keywords:** relapsing multiple sclerosis, fingolimod, disease-modifying therapy, lymphoblastic leukemia

## Abstract

A man with relapsing multiple sclerosis, treated with fingolimod 0.5 mg/d for 15 months, developed acute lymphoblastic leukemia and died 4 months after immune ablation and bone marrow allograft, from graft versus host disease. To our knowledge, this is the first case of acute lymphoblastic leukemia reported in a patient treated with fingolimod. Although no causal relationship can be established between fingolimod use and acute lymphoblastic leukemia risk in this single case, future surveillance for lymphatic cell malignancies in patients treated with fingolimod appears justified.

## Introduction

Fingolimod (FGM) is a sphingosine-1-phosphate (S1P) receptor modulator that has been approved for the treatment of relapsing multiple sclerosis (RMS). When compared with placebo-treated patients, FGM reduces risks of relapse, sustained disability progression, and disease burden increase on brain magnetic resonance imaging.^1^ Among its biological actions, FGM sequesters immune-reactive T- and B-cell lymphocytes in peripheral lymphoid organs.^2^ Because naïve and central memory cells are selectively retained,^3^ this potential impact of FGM treatment on adaptive immunity may alter susceptibility to opportunistic infections and neoplasia. This report presents a patient who developed acute lymphoblastic leukemia (ALL) while being treated with FGM for RMS.

## Case

The patient was a previously healthy 34-year-old man, with no known cancer risk, who experienced acute bilateral hand tingling and urinary incontinence in 2006, which spontaneously resolved after several weeks, transient numbness and weakness in his right hand in January 2009, and left hemiparesis in June 2009. Brain magnetic resonance imaging in 2009 revealed multiple cerebral hemispheric white matter T2 lesions and a gadolinium-enhancing lesion in the pons. Cerebral spinal fluid examination, performed at another institution, revealed elevated IgG index and positive oligoclonal bands. Treatment with intramuscular (IM) interferon β-1a (IFNβ-1a), 30 µg weekly, was started June 2009, and he remained clinically stable until May 2010 when he developed right retro-orbital pain and painful paresthesias of his feet. He suffered subsequent relapses in August, October, and December 2010, the details of which were not further described in his outside records. He remained on IM IFNβ-1a until January 2011, when he was switched to oral FGM 0.5 mg daily. FGM was discontinued June 2011 due to low absolute lymphocyte count (<200/mL) and a CD4+ lymphocyte count of 15. Because of increased right-sided weakness, FGM was resumed September 2011. Following a detailed review of the patient’s medication record with his previous treating neurologist, IFNβ-1a was the only agent employed to treat his RMS between June 2009 and January 2011, and at no time did the patient receive mitoxantrone. On December 6, 2012, he presented with deep popliteal and sural vein thromboses, and a peripheral white blood cell count >100 000, 80% blast cells. Biopsy revealed hypercellular bone marrow, 90% blast cells, with pre-B cell ALL, expressing CD19, CD22, CD34, and a subset positive for CD15. Patient was Ph1 negative, and fluorescence in situ hybridization revealed mixed lineage leukemia (11 q23) rearrangement. Following treatment with methotrexate, methylprednisolone, leucovorin, and cytarabine, he underwent an unrelated allogeneic bone marrow transplantation in March 2013, but developed graft-versus-host disease, with resultant encephalopathy, renal and hepatic failure, extensive desquamation of skin, and died April 2013.

## Discussion

We describe the case of a man with RMS and being treated with FGM, without known risk factors for, or family history of leukemia or other lymphoproliferative disorders, who developed ALL. To our knowledge, this is the first known case of leukemia reported in a human receiving FGM.

Although FGM has been used experimentally to treat B-cell lymphoma and suppressed ALL allografts in mice,^4^ at FGM doses higher than the comparable recommended human dose, there was increased risk of malignant lymphoma.^5^ FGM has also been demonstrated to destroy acute lymphoblastic leukemia cells, including human ALL cells ex vivo.^6-8^

Although no prior cases of leukemia in humans treated with FGM were found in the PubMed database, 1 case of diffuse B-cell lymphoma, 2 cases of cutaneous T-cell lymphoma and 1 case of lymphatoid papulosis were reported in pre-marketing clinical trials of FGM as of May, 2012.^5^

This patient developed an 11q23 mixed lineage leukemia, which is interesting, given that he had not been treated with mitoxantrone, and the lack of evidence that FGM has effect on topoisomerase I or II. Though lymphomas may be a general effect of immune suppressive therapy, pre-B ALL is not expected to result from chronic immunosuppression, suggesting that the etiology of ALL is by an entirely different mechanism.

Fingolimod is known to cause lymphopenia from altered lymphocyte trafficking via interaction with sphingosin-1-phosphate (S1P) receptors, rather than depletion of total body immune cells. The downstream signaling component of the S1P pathway^9,10^ includes Bruton tyrosine kinase (BTK), β-arrestin 2, lipopolysaccharide-responsive beige-like anchor protein, dedicator of cytokinesis 8, and Wiskott-Aldrich syndrome protein. One might speculate that there is a potential leukemogenic effect of modulation of the S1P pathway by FGM.

Given an ALL incidence of more than 6,000 cases in the United States in 2013,^11^ it is unwarranted to attribute this single case of ALL to FGM use. However, given the potential direct effect of FGM on lymphatic cells via the S1P pathway, the possibility that FGM could increase ALL risk should not be dismissed. This case highlights a potential class effect risk for this and similar immune-modulators. Thus, we believe it is important that physicians be aware of this case and the possible risk of developing acute leukemia in patients treated with FGM.
